# Experimental and Theoretical Study of the Enthalpy of Formation of 3,6-Diphenyl-1,2,4,5-Tetroxane Molecule

**DOI:** 10.1100/tsw.2002.126

**Published:** 2002-02-15

**Authors:** N.L. Jorge, L.C.A. Leiva, M.G. Castellanos, M.E. Gomez Vara, L.F.R. Cafferata, E.A. Castro

**Affiliations:** Area de Fisicoquímica, FACENA, Universidad Nacional del Nordeste, Avda. Libertad 5300, 3400 Corrientes, Argentina; LADECOR, Departamento de Química, Facultad de Ciencias Exactas, Universidad, Nacional de La Plata, Calles 47 y 115, La Plata 1900, Argentina; CEQUINOR, Departamento de Química, Facultad de Ciencias Exactas, Universidad, Nacional de La Plata, Calles 47 y 115, C.C. 962, La Plata 1900, Argentina

**Keywords:** 3, experimental, enthalpy of formation, semiempirical calculation, ab initio calculation

## Abstract

We report the results obtained for the experimental determination and the theoretical calculation of the enthalpy of formation of 3,6-diphenyl-1,2,4,5-tetroxane molecule. The experimental work was performed using a macrocalorimeter to measure the combustion heat, and the sublimation enthalpy was determined via the measurement of the vapor pressure at equilibrium with the vapor phase at different temperatures resorting to the Clapeyron-Claussius equation. Theoretical calculations were performed using semiempirical AM1 and PM3 methods as well as *ab initio* techniques at the 3-21, 6-31G(d,p), and 6-311G(d,p) basis set levels.

## INTRODUCTION

Peroxides are widely used in several chemistry areas due to their high reactivity with organic and inorganic substrates[1]. Their specific properties as free radicals reaction initiators are amply employed in synthetic chemistry[2], especially in polymerization processes. Unfortunately, several members of these kind of compounds are very unstable and just a small number are easy to manipulate with a sensible degree of security and are available as commercial products.

Among these peroxides is a large number of peroxyacids and peroxyesters[3]. Although the importance of these compounds is well known in the chemistry area, several structural aspects and reactivity modeling need to be precisely defined[4]. Chemical reactivity of these molecules, in thermal decomposition or induced by metals through an electronic-transfer process decomposition, probably involves an homolytic breakdown of the O–O bond with free radicals formation as a critical reaction step[5,6]. These chemical species, or their decomposition products, are normally involved in quick reactions with several substrates[2]. Therefore, it is necessary to know the tentative schema of the reactivity models of peroxyacids and peroxyesters as well as the energies associated with the homolytic rupture of these molecules and their corresponding radicals. These parameters represent basic information about the chemical reactivity of these molecules, although the subsequent reactions involving the radicals (usually with very large rate constants) could determine the reaction paths. Energies of the homolytic breakdown (dissociation) are known for a small number of peroxides and standard literature registers just a rather limited number of confident information for radicals[7,8]. These values are known with an accuracy of c.a. ±1 kcal/mol due to experimental difficulties[9,10].

The dissociation enthalpy ΔH can be calculated via the enthalpy of formation $\Delta H_f$ of the molecules involved, using Eq. 1:

$$\Delta H(R - R) = \Delta H_f(R) + \Delta H_f(R) - \Delta H_f(R - R) \qquad (1)$$

The enthalpy of formation for molecules appearing in Eq. 1 is known.

The aim of this paper is to report the experimental enthalpy of formation of the 3,3,6,6-tetramethyl 3,6-diphenyl-1,2,4,5-tetroxane molecule (DPhTOx), as well as the results calculated through theoretical, semiempirical, and *ab initio* methods.

## EXPERIMENTAL METHOD

A macrocalorimeter has been used to measure the combustion heat[11]. Substance was located in a gelatine capsule and was burnt in an oxygen atmosphere at P = 25 atm. The necessary current for ignition is 2 amperes (amp). The water equivalent E of the calorimeter was determined with a reference substance (benzoic acid) and the experimental average value is E = 670.0 ± 1.5 cal/grade.

A summary information of the auxiliary quantities for the combustion heat experiments and complementary details are given in Table 1, where we have included the standard deviations for each experimental result. Combustion enthalpies and formation heats are given in Table 2.

Sublimation enthalpy of DPhTOx was determined via the vapor pressure at equilibrium with the vapor phase at different temperatures resorting to the Claussius-Clapeyron equation. Measurements were made with the mercury manometer through a Bodestein differential equipment. For each temperature of the substance we determined the equilibrium vapor pressure. Experimental values are displayed in Table 3.

## RESULTS

### Thermochemical Measurements

The determination of the combustion heat was made in a macrocalorimeter (Malher adiabatic calorimetric bomb)[11]. Substance was burnt in an oxygen atmosphere at P = 25 atm. Ignition current was 2 amperes. The equivalent energy of the calorimeter E was determined with a standard reference sample of benzoic acid and the measured E was 670.0 ± 1.9 cal/K, for every experiment. The crystalline compound under study was pressed in a gelatine capsule. The isothermal process at the bomb and the corresponding correction to the standard normal state was made following the procedure described by Groth[12] (Table 2). Results obtained in each experimental experience are listed in Table 1. The specific combustion heats ($\Delta U_c$) obtained in

**TABLE 1**
**Typical Results of the Combustion Experiences**

| | 1 | 2 | 3 | 4 | 5 | 6 |
|---|---|---|---|---|---|---|
| **$M_{DFT}$/g** | 0.0820 | 0.0894 | 0.0800 | 0.0851 | 0.0325 | 0.0444 |
| **$m_{gel}$/g** | 0.3562 | 0.2786 | 0.3428 | 0.3567 | 0.1280 | 0.1282 |
| **$m_{Fe}$/g** | 0.0428 | 0.0429 | 0.0447 | 0.0428 | 0.0385 | 0.0341 |
| **ΔT/K** | 0.66043 | 0.57478 | 0.63960 | 0.66765 | 0.25475 | 0.27831 |
| **($m_{agua}$+E) ΔT/cal[a]** | 2222.65 | 1937.01 | 2155.45 | 2249.98 | 858.51 | 937.91 |
| **$m_{gel}$ $\Delta U_{gel}$/cal[b]** | 1568.70 | 1226.95 | 1509.69 | 1570.91 | 563.71 | 564.59 |
| **$m_{Fe}$ $\Delta U_{Fe}$/cal[c]** | 68.48 | 68.64 | 71.52 | 68.48 | 61.66 | 54.57 |
| **$\Delta U_c$/cal/g** | 7176.39 | 7174.66 | 7178.01 | 7175.01 | 7175.25 | 7179.10 |
| **$\Delta H_c$/cal/mol** | -1750.45 | -1750.02 | -1750.84 | -1750.11 | -1750.17 | -1751.11 |

**TABLE 2**
**Specific Combustion Energies ($\Delta U_c$) Resulting from the Combustion Experiences in (cal/grade) and Normal Combustion Enthalpies ($\Delta H^o_c$) in kcal/mol**

| Experiment # | $\Delta U_c$(c) | $\Delta H^o_c$(c) |
|---|---|---|
| **1** | 7176.39 | -1750.45 |
| **2** | 7174.66 | -1750.02 |
| **3** | 7178.01 | -1750.84 |
| **4** | 7175.01 | -1750.11 |
| **5** | 7175.25 | -1750.17 |
| **6** | 7179.10 | -1751.11 |
| **Mean** | 7176.40 | -1750.45 |
| **SDa** | ±2.0 | ±0.3 |

every combustion determination are given in Table 2. Normal enthalpies of combustion ($\Delta H_c$) and the normal heat of formation ($\Delta H_f$) calculated from the previous data are presented in Table 3.

In order to reduce data to the normal conditions, we employed the conventional procedures. We have also calculated the sublimation heat of DPhTOx from the vapor pressure equilibrium with the vapor phase at different temperatures (Table 4) using the Claussius-Clapeyron equation. Measurements were performed resorting to a Bodestein differential manometer.

Typical results for the experimental combustion determinations of diperoxide in study are given in Table 1. Individual values of combustion energies ($\Delta U_c$), together with the average value of experimental determinations and its standard deviation are displayed in Table 2. In order to obtain the normal enthalpies of formation ($\Delta H^o_f$)from the normal combustion enthalpies ($\Delta H^o c$) we used the following molar enthalpies of formation[12]: $H_2O$(l) = – (68.38 ± 0.04) kcal/mol, $CO_2$(g) = – (94.14 ± 0.13) kcal/mol.

In Table 3 we list the normal molar enthalpies of combustion and normal molar enthalpies of formation in the gas and solid states. The normal deviations of the average values include the

**TABLE 3**
**Molar Enthalpies of DPhTOx (kcal/mol)**

| ***Experiment #*** | $\Delta H_c^0$ **(c)** | $\Delta H_f^0$ **(c)** | $\Delta H_f^0$ **(g)**[a] |
|---|---|---|---|
| **1** | -1750.45 | 23.82 | 15.62 |
| **2** | -1750.02 | 23.39 | 15.19 |
| **3** | -1750.84 | 24.21 | 16.01 |
| **4** | -1750.11 | 23.48 | 15.28 |
| **5** | -1750.17 | 23.54 | 15.34 |
| **6** | -1751.11 | 24.48 | 16.28 |
| **Mean** | -1750.45 | 23.82 | 15.62 |
| **SD** | ±0.3 | ±0.3 | ±0.3 |

[a] Standard enthalpies of formation in gas the phase obtained from the sublimation enthalpies ($\Delta H_s$ = 8.2 kcal/mol) determined from the vapor pressure measurements shown in Table 4.

**TABLE 4**
**Vapor Pressure of DPhTOx at Different Temperatures, Measured via a Bodestein Differential Manometer**

| **T(C)** | **p(mmHg)** |
|---|---|
| 18.0 | 12.0 |
| 29.0 | 2.0 |
| 34.8 | 2.0 |
| 41.7 | 36.6 |
| 50.2 | 50.0 |
| 58.6 | 68.0 |

calibration uncertainties and those associated to the combustion energies of the auxiliary materials. Finally, we determine the thermochemical properties of DPhTOx.

## Theoretical Calculations

One of the main purposes of the calorimetric studies is to find the energies of the substituted compounds. Thermochemical relationships are important since in some cases the formation enthalpy, for example those corresponding to hydrocarbons, are know with great accuracy, but enthalpies of formation of substituted compounds usually are known with low degree of accuracy, or they are doubtful or even completely inaccessible. A way to know the energetic effects when substituents are introduced is to employ theoretical methods.

## METHODOLOGY

In order to make the theoretical calculation of the enthalpy of formation, it is necessary to find out a suitable isodesmic chemical reaction to optimize the corresponding molecular structures and to perform the vibrational frequencies (using the optimized geometries), applying the method to obtain the total molecular electronic energy at 298 K.

We used the Gaussian 94 package to perform the calculations[13]. When one tries to optimize molecular geometries, it is necessary to locate the absolute minimum point over the potential energy hypersurface which gives the corresponding equilibrium structure. The optimization is complete when the numerical process converges, i.e., when forces are zero. The convergence criteria employed in the Gaussian 94 package are:

- Forces are practically zero.
- The average square root of forces must be zero.
- The shift calculation for the next step must be small.
- Second derivative of the shift must be zero.

## RESULTS AND DISCUSSION

The isodesmic reaction to calculate the heat of formation is:

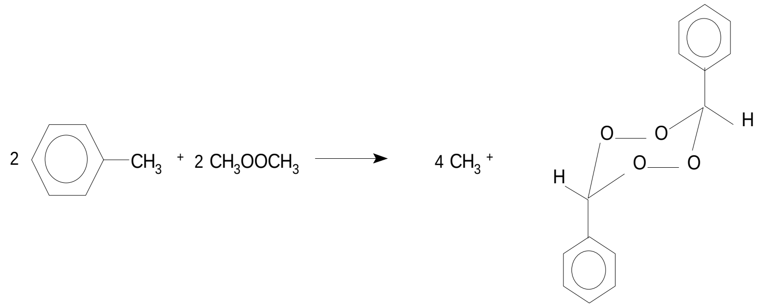

We made a geometric optimization procedure for each compound involved in the chemical reaction using semiempirical methods (AM1 and PM3, respectively) and *ab initio* techniques: at the 3-21G, 6-31G(d,p) and 6-311G(d,p) basis set levels. In Table 5 we show the energy values for each of them.

**TABLE 5**
**Calculated Energies of Formation (in Hartree Units) and Heat of Reaction (in Hartree Units)**

| | AM1 | PM3 | RHF 3-21G | RHF 6-31G(d,p) | RHF 6-3111G(d,p) |
|---|---|---|---|---|---|
| **$PhCH_3$** | 0.159098 | 0.155271 | –268.095478 | –269.611640 | –269.658609 |
| **$CH_4$** | 0.034185 | 0.028432 | –39.925088 | –40.150502 | –40158264 |
| **$CH_3OOCH_3$** | 0.047722 | 0.032618 | –227.484385 | –228.742221 | –228.699771 |
| **DFT** | 0.298213 | 0.262975 | –831.503374 | –836.139541 | –836.310114 |
| **$\Delta H_r$** | 0.021313 | 0.000925 | –0.044000 | –0.033827 | –0.030850 |
| **$\Delta H_f$(kcal/mol)** | 48.70 | 34.90 | 7.87 | 14.25 | 16.12 |

**TABLE 6**
**Normal Energy of Formation in kcal/mol**

| | AM1 | PM3 | RHF 3-21G | RHF 6-31G(d;p) | RHF 6-311G(d;p) |
|---|---|---|---|---|---|
| DFT | 48.70 | 34.90 | 7.87 | 14.25 | 16.12 |

In Table 6 we give the values obtained from the normal energies of formation of DFT, employing the data corresponding to each molecule of the reaction scheme.

## CONCLUSION

The experimental determination of heats of formation by means of the combustion heat data is a rather exact method, and it is relatively easy to perform it in a laboratory. The theoretical procedure based on using the isodesmic chemical reaction allows one to obtain theoretical data, which usually are very close to experimental data.

Theoretical calculations made at the *ab initio* level with a 6-311G(d,p) basis set is quite similar to the experimental determination. Geometrical parameters of the DFT molecule derived from this calculation reproduce quite well the crystallographic data reported by the literature[12].
